# Opposing Effects of ApoE2 and ApoE4 on Glycolytic Metabolism in Neuronal Aging Supports a Warburg Neuroprotective Cascade against Alzheimer’s Disease

**DOI:** 10.3390/cells12030410

**Published:** 2023-01-25

**Authors:** Xin Zhang, Long Wu, Russell H. Swerdlow, Liqin Zhao

**Affiliations:** 1 Department of Pharmacology and Toxicology, School of Pharmacy, University of Kansas, Lawrence, KS 66045, USA; 2 Department of Neurology, University of Kansas Medical Center, Kansas City, KS 66160, USA; 3 Neuroscience Graduate Program, University of Kansas, Lawrence, KS 66045, USA

**Keywords:** late-onset Alzheimer’s disease (LOAD), apolipoprotein E, ApoE2, ApoE4, hexokinase, glycolysis, Warburg effect, brain resilience

## Abstract

Apolipoprotein E4 (ApoE4) is the most recognized genetic risk factor for late-onset Alzheimer’s disease (LOAD), whereas ApoE2 reduces the risk for LOAD. The underlying mechanisms are unclear but may include effects on brain energy metabolism. Here, we used neuro-2a (N2a) cells that stably express human ApoE isoforms (N2a-hApoE), differentiated N2a-hApoE neuronal cells, and humanized ApoE knock-in mouse models to investigate relationships among ApoE isoforms, glycolytic metabolism, and neuronal health and aging. ApoE2-expressing cells retained robust hexokinase (HK) expression and glycolytic activity, whereas these endpoints progressively declined with aging in ApoE4-expressing cells. These divergent ApoE2 and ApoE4 effects on glycolysis directly correlated with markers of cellular wellness. Moreover, ApoE4-expressing cells upregulated phosphofructokinase and pyruvate kinase with the apparent intent of compensating for the HK-dependent glycolysis reduction. The introduction of ApoE2 increased HK levels and glycolysis flux in ApoE4 cells. PI3K/Akt signaling was distinctively regulated by ApoE isoforms but was only partially responsible for the ApoE-mediated effects on HK. Collectively, our findings indicate that human ApoE isoforms differentially modulate neuronal glycolysis through HK regulation, with ApoE2 upregulating and ApoE4 downregulating, which markedly impacts neuronal health during aging. These findings lend compelling support to the emerging inverse-Warburg theory of AD and highlight a therapeutic opportunity for bolstering brain glycolytic resilience to prevent and treat AD.

## 1. Introduction

To date, the molecular pathogenesis for most Alzheimer’s disease (AD) cases is unknown, and no effective treatment exists. For over three decades, the accumulation of amyloid beta (Aβ) was considered by many to serve as the initiating trigger. However, the failure of numerous Aβ-targeted interventions questions the validity of such therapeutic approaches and stresses the need to broaden the scope in search of new paths to the prevention and treatment of AD.

The presence of an apolipoprotein E4 (ApoE4) allele increases the risk of developing late-onset AD (LOAD). In the brain, ApoE is predominantly produced by astrocytes, while under certain circumstances, such as traumatic brain injury, neurons will also synthesize and secrete ApoE [1]. ApoE transports lipids, promotes neurite outgrowth, and facilitates neuronal communication [2]. ApoE exists in three major isoforms conferring profoundly distinct influences in AD etiology. ApoE2 is a rare variant present in approximately eight percent of the general population and is viewed as an exceptionally neuroprotective variant against AD. ApoE3, the most common form represented in 80% of the population, confers neutral risk. Although ApoE4 only occurs in 14% of the population, it is present in nearly 40~65% of AD cases [3,4]. ApoE4 contributes to an earlier age of onset of AD, more rapid disease progression, greater cognitive dysfunction, and altered responses to AD treatments. ApoE2 mitigates age-related cognitive changes and associates with reduced Aβ deposition and fewer neurofibrillary tangles [5,6,7]. A better understanding of the biological bases underlying the neuroprotective versus neurodegenerative properties of ApoE isoforms could help illuminate AD pathophysiology and enable the discovery of novel therapeutic targets.

Accumulating evidence demonstrates that reduced brain glucose utilization occurs in early AD, particularly during the preclinical phase [8,9,10,11], which could reflect an impaired glycolytic metabolism [12,13,14,15]. A recent study reported that in AD brains, glucose levels positively correlate, whereas glycolysis flux negatively correlates with plaque and tangle deposition [12]. Impaired glycolysis promotes tauopathy in individuals with cerebral amyloid [13]. Conversely, robust neuronal glycolysis blunts Aβ toxicity [16]. Moreover, in cognitively intact ApoE4 carriers, the cerebral metabolic rate of glucose is reduced in the same regions as found in clinically affected AD patients [17,18]. Furthermore, the activation of aerobic glycolysis is shown to play a key role in spatial memory acquisition in mice [19]. Our lab recently reported that, compared to mice that harbor ApoE3 or ApoE4 transgenes, the brains of ApoE2-bearing mice exhibit increased hexokinase (HK) protein expression and enzymatic activity, basal and maximum glycolysis flux, and ATP production [20]. These findings suggest that the ApoE genetic status modulates brain glycolysis; however, its functional significance, particularly in neuronal aging, remains to be determined.

In the present study, we used neuro-2a (N2a)-hApoE stable cell lines, retinoic acid (RA)-induced differentiated N2a-hApoE neuronal cells, and humanized ApoE knock-in mouse models to determine the interactive impact of ApoE and aging on neuronal glycolytic metabolism and on overall health. We found that as cells age, ApoE2-expressing cells retained robust HK protein expression and glycolytic activity; in stark contrast, ApoE4-expressing cells exhibited a progressive decline in both HK expression and glycolysis flux. Changes in HK and glycolytic status paralleled changes in the general state of cellular wellness evidenced by cellular appearance and metabolic activity. Introducing ApoE2 into ApoE4 cells mitigated ApoE4-associated HK and glycolytic deficits. Our findings suggest a potential neuroprotective role for ApoE2 that may arise through the upregulation of neuronal HK and glycolysis flux. Based on these findings, we propose that ApoE isoforms impact AD via a Warburg or inverse-Warburg mechanism, and a therapeutic approach that promotes a Warburg phenotype may represent a new promising opportunity for preventing and treating AD.

## 2. Materials and Methods

### 2.1. Cell Models

#### 2.1.1. Generation and Maintenance of N2a-hApoE Stable Cell Lines

Mouse neuro-2a (N2a) cells that stably express human ApoE isoforms (N2a-hApoE) were obtained as previously described [21]. Briefly, 2 μg of human pCMV-APOE ε2, pCMV-APOE ε3, or pCMV-APOE ε4 plasmid were transfected using Lipofectamine 3000. After 48 h, the medium was replaced and supplemented with Geneticin (G418, Thermo Fisher Scientific, Waltham, MA, USA). Cells were maintained and subjected to serial dilutions in 96-well plates. Approximately twenty to thirty wells of each ApoE genotype that contained only one cell were numbered and considered as the monoclonal colony. Cells were maintained in Dulbecco’s Modified Eagle Medium (DMEM, high glucose) supplemented with 10% fetal bovine serum (FBS, Thermo Fisher Scientific) containing 600 μg/mL G418 in a humidified incubator under an atmosphere of 5% CO_2_ at 37 °C.

#### 2.1.2. Neuronal Differentiation of N2a-hApoE Stable Cell Lines with Retinoic Acid

The N2a cell line differentiated with retinoic acid (RA) is widely used as a neuronal cell model [22,23]. N2a-hApoE stable cell lines were seeded in 6-well plates at a cell density of 1 × 10^5^/mL in DMEM supplemented with 2% FBS and containing 20 μM all-trans-RA (Sigma-Aldrich, Inc., St. Louis, MO, USA). The medium was replaced with fresh medium containing RA every 2 days. After 4 days, differentiated neuron-like N2a-hApoE cells (dN2a-hApoE) were proceeded to further studies.

### 2.2. Animals

Animal Use Statement (AUS # 220-08) was approved by the IACUC at the University of Kansas on 10 January 2019. Six-month-old ApoE2, ApoE3, and ApoE4 gene-targeted replacement (ApoE2-TR, ApoE3-TR, and ApoE4-TR) female mice were obtained from Taconic [20]. These ApoE-TR mice express human ApoE2, ApoE3 or ApoE4 under the control of the endogenous mouse promoter. Glial cells express human ApoE in a native conformation at physiologically regulated levels, and in the same temporal and spatial pattern as endogenous mouse ApoE [24]. The humanized ApoE knock-in (ApoEKI) mice were purchased from the Jackson Laboratory (stock #029018, #027894) (Bar Harbor, ME, USA). These ApoEKI mice express ApoE isoforms from the endogenous ApoE locus at a physiological level with fewer breeding restrictions [25]. Mice were bred and housed until reaching 6–7 months old. Both female and male ApoEKI mice were used. Mice were euthanized through carbon dioxide inhalation according to protocol. Cortical tissues were harvested and stored at −80 °C.

### 2.3. Western Immunoblotting

Cells were rinsed with cold phosphate-buffered saline (pH 7.4, Thermo Fisher Scientific) and lysed in neuronal protein extraction reagent (NPER) containing protease and phosphatase inhibitors (PPI) (Thermo Fisher Scientific) for 10–15 min on ice. Whole-cell lysate was centrifuged at 4 °C for five minutes at 1500× *g*. The supernatant was collected. Mouse cortical tissues were lysed using Tissue Protein Extraction Reagent (T-PER) (Thermo Fisher Scientific) containing PPI. Cortex lysate was collected after centrifuge at 12,000× *g* RPM for 8 min at 4 °C. The protein concentration was determined by the BCA protein assay kit (Thermo Fisher Scientific). Samples were then diluted in Laemmli sample buffer (Bio-Rad, Hercules, CA, USA) with 2-mercaptoethanol (Bio-Rad) and boiled at 95 °C for 5 min. An equal amount of total protein was separated by 10% sodium dodecyl sulfate-polyacrylamide gel electrophoresis (SDS-PAGE), then transferred onto 0.2 μm pore-sized PVDF membranes (Bio-Rad). The membrane was blocked with 5% non-fat milk in TBS (100 mL 10 × TBS (200 mM Tris, 1.5 M NaCl), 900 mL ddH_2_O, pH 7.6) for one hour at room temperature, followed by incubation with primary antibody at 4 °C overnight. The membranes were then washed with TBST (100 mL 10 × TBS (200 mM Tris, 1.5 M NaCl), 10 mL 10% Tween-20, 890 mL ddH_2_O, pH 7.6), followed by hybridization with the horseradish peroxidase (HRP)-conjugated secondary antibody. After washing with TBST again, the bands on the membrane were detected by C-Digit Blot Scanner (LI-COR, Lincoln, NE, USA) after applying the enhanced chemiluminescence (ECL) reagent (Bio-Rad). Quantification was obtained using the Image Studio Version 4.0 imaging digitizing software, standardized by the internal loading control protein. The following primary antibodies were applied: goat anti-Apolipoprotein E (1:4000, EMD Millipore, Burlington, MA, USA), rabbit anti-hexokinase I (1:2000, Cell Signaling Technology, Danvers, MA, USA), rabbit anti-hexokinase II (1:1500, Cell Signaling Technology), mouse anti-GAPDH (1:6000, Santa Cruz Biotechnology, Inc., Dallas, TX, USA), mouse anti-PFKP (1:600, Santa Cruz Biotechnology, Inc), rabbit anti-Phospho-Akt (Ser473) (1:1000, Cell Signaling Technology), rabbit anti-Akt (pan) (1:1000, Cell Signaling Technology), rabbit anti-PKM1 (1:1000, Cell Signaling Technology), rabbit anti-GLUT1 (1:1000, Sigma-Aldrich), mouse anti-GLUT3 (1:1000, Santa Cruz Biotechnology, Inc.), rabbit anti-GLUT-4 (1:1000, EMD Millipore), rabbit anti-β-actin (1:5000, Thermo Fisher Scientific), HRP anti-β-actin (1:5000, BioLegend, San Diego, CA, USA), and mouse anti-β-tubulin (1:5000, Thermo Fisher Scientific). The secondary antibodies included goat anti-Mouse, HRP (1:5000, Thermo Fisher Scientific), goat anti-rabbit, HRP (1:5000, Thermo Fisher Scientific), and rabbit anti-goat, HRP (1:3000, Thermo Fisher Scientific).

### 2.4. Hexokinase Activity Assay

The hexokinase activity assay was conducted as previously described [21]. Briefly, cell lysates were harvested as described above. Hexokinase activity was measured as the total glucose phosphorylating capacity of the lysate, based upon the reduction of NAD^+^ through a coupled reaction with glucose-6-phosphate dehydrogenase. Results were measured spectrophotometrically by monitoring the increase in absorbance at 340 nm every 1 min for 30 min under 37 °C. The initial linear slope of curve was used to determine ΔA/min for further calculation. Hexokinase assay solution was prepared with 13.3 mM MgCl2, 0.112 M glucose, 0.55 mM adenosine 5′ triphosphate, 0.227 mM NAD^+^, and 1 IU/mL glucose-6-phosphate dehydrogenase in 0.05 M Tris-HCl buffer, pH 8.0 as described previously [26]. After 6–8 min of incubation at room temperature to reach equilibrium, 15–20 μg cell extracts were added into 150 μL assay solution to initiate the reaction. Results were normalized to total cellular protein content using the BCA assay.

### 2.5. Glycolytic Stress Test

Extracellular flux (XF) analysis (Agilent Technologies) was used to evaluate the glycolytic activity. Basal glycolysis and glycolytic capacity were measured by assessing the extracellular acidification rate (ECAR). Cells expressing human ApoE isoforms were seeded at a density of 8000–10,000 cells/well on Seahorse XF96 culture plates. On the experiment day, glycolytic stress test assay medium was prepared as XF base medium (Agilent Technologies, Santa Clara, CA, USA) containing 2 mM glutamine, pH 7.4. The assay was initiated by replacing the growth medium with pre-warmed assay medium and incubating the cell culture plate at 37 °C without CO_2_ at least 1 h prior to the measurement. Glycolytic parameters of human ApoE2/3/4-expressing cells were then measured using the Seahorse XF Analyzer following successive injections of 10 mM glucose, 2 μM oligomycin, and 50 mM 2-Deoxy-D-glucose to each well. Cell culture plates were saved after the assay and used for the normalization of the results by assessing the protein content in each well.

### 2.6. LIVE/DEAD Cell Assay

N2a-hApoE cells were seeded on ibiTreat #1.5 polymer coverslip μ-Slide (Ibidi, Martinsried, Germany) with complete growth medium. The medium was replaced with fresh medium containing G418 every two days. On day 4, the culture medium was removed carefully and replaced with the LIVE/DEAD cell assay reagents (Thermo Fisher Scientific) at the working concentration, together with Hoechst dye. 10× images were obtained under the Leica DMI4000 B inverted microscope with FITC (495–519 nm) and Texas Red (595–605 nm) filters. 10–15 images were obtained for each isoform in each passage with similar cell density. Hoechst staining and dead cells were used as controls.

### 2.7. Phase Contrast Imaging

Cellular appearances were evaluated and compared using phase contrast imaging. N2a-hApoE cells were seeded in 35 mm glass-bottom poly-D-lysine pre-coated culture dishes (MatTek Corporation, Ashland, MA, USA). Cells were maintained for 4 days and were replaced with fresh medium every 2 days. Phase contrast images were acquired using an Olympus IX81 inverted epifluorescence microscope equipped with a cage incubator for maintaining precise temperature, humidity and CO_2_ control. Slidebook Software Version 6.0 was used for microscope control, image acquisition, image processing, and data analysis (Intelligent Imaging Innovations, Inc., Denver, CO, USA). Images were acquired using a 40× phase contrast magnification on day 4 under 37 °C and 5% CO_2_.

### 2.8. Metabolic Activity Assay

A cell permeable resazurin-based solution, PrestoBlue^®^ (Invitrogen Life Technologies, Carlsbad, CA, USA), which measures the reducing power of viable cells, was used as an indicator of cellular metabolism. 10 µL of PrestoBlue reagent was directly added to cells in 90 µL of culture medium in a 96-well plate. The plate was incubated at 37 °C for 1 h and fluorescence was read at Ex/Em 528/20,600/40 nm using a plate reader (BioTek). Wells containing only cell culture media (no cells) were used as background control wells. Results were normalized to protein content of each well. Higher fluorescence units indicate greater metabolic activity.

### 2.9. Transfection of ApoE2 in N2a-ApoE4 Cells

Human ApoE2 cDNA clones expressed in the mammalian vector pCMV6 with C-terminal MycDDK Tag were obtained as previously described [20]. An empty pCMV6 vector was used in the control group. Briefly, 2 µg human ApoE2 cDNA was transfected to N2a cells stably expressing human ApoE4 using jetPRIME^®^ transfection reagent according to the manufacturer manual. Fresh complete medium was replaced 4 h after transfection. Cells were then continuously incubated for 48–72 h before proceeding to further experiments.

### 2.10. Protein Carbonylation Assay

Protein carbonylation levels in mouse cortex lysates were measured using the OxiSelect Protein Carbonyl Immunoblot Kit (Cell Biolabs Inc., San Diego, CA, USA) according to the manufacturer’s instructions with minor modifications. Cortex lysate samples were obtained as described in the Western Immunoblotting method section. Briefly, 20 μg protein of each sample was loaded and separated on NuPAGE^TM^ 4–12% Bis-Tris Gel (Thermo Fisher Scientific), followed by transferring onto 0.2 μm pore-sized PVDF membranes (Bio-Rad). After transferring, 2,4-dinitrophenylhydrazine (DNPH) derivatization was conducted as follows: membranes were immersed in 100% methanol for 15 s, dried out for 5 min at room temperature, equilibrated in 1 × TBS containing 20% methanol for 5 min, washed with 2N HCl for another 5 min, and then incubated in 1 × DNPH for exactly 5 min followed by 2N HCl washing for three times. After DNPH derivatization, membranes were washed five times in 100% methanol, 5 min each. Blocking was performed by incubating the DNPH-treated membranes with 5% non-fat milk in 1 × TBS for 1 h at room temperature. Membranes were washed with 1 × TBST three times before incubating with Rabbit Anti-DNP antibody in 1% non-fat milk overnight at 4 °C (anti-DNP 1:1000, Cell Biolabs Inc.). On the next day, after washing with 1 × TBST three times, the membranes were incubated with the secondary antibody in 5% non-fat milk for one hour at room temperature, followed by another four times of 1 × TBST washing. Densitometry of the immunolabeling was measured using C-Digit Blot Scanner (LI-COR) after ECL application. Results were normalized to internal loading control protein, β-actin.

### 2.11. Statistical Analyses

GraphPad Prism 6 (GraphPad Software, La Jolla, CA, USA) was used in statistical analyses. Data represent the group mean ± SEM. Group comparisons were analyzed by one-way analysis of variance (ANOVA) with Tukey’s post hoc test or Student’s *t*-test. A *p* value lower than 0.05 was considered statistically significant. 

## 3. Results

### 3.1. HK Expression Remained Relatively Stable in ApoE2- and ApoE3-Expressing Cells but Exhibited a Gradual Decrease with Increasing Passages in ApoE4-Expressing Cells

**Figure 1:**
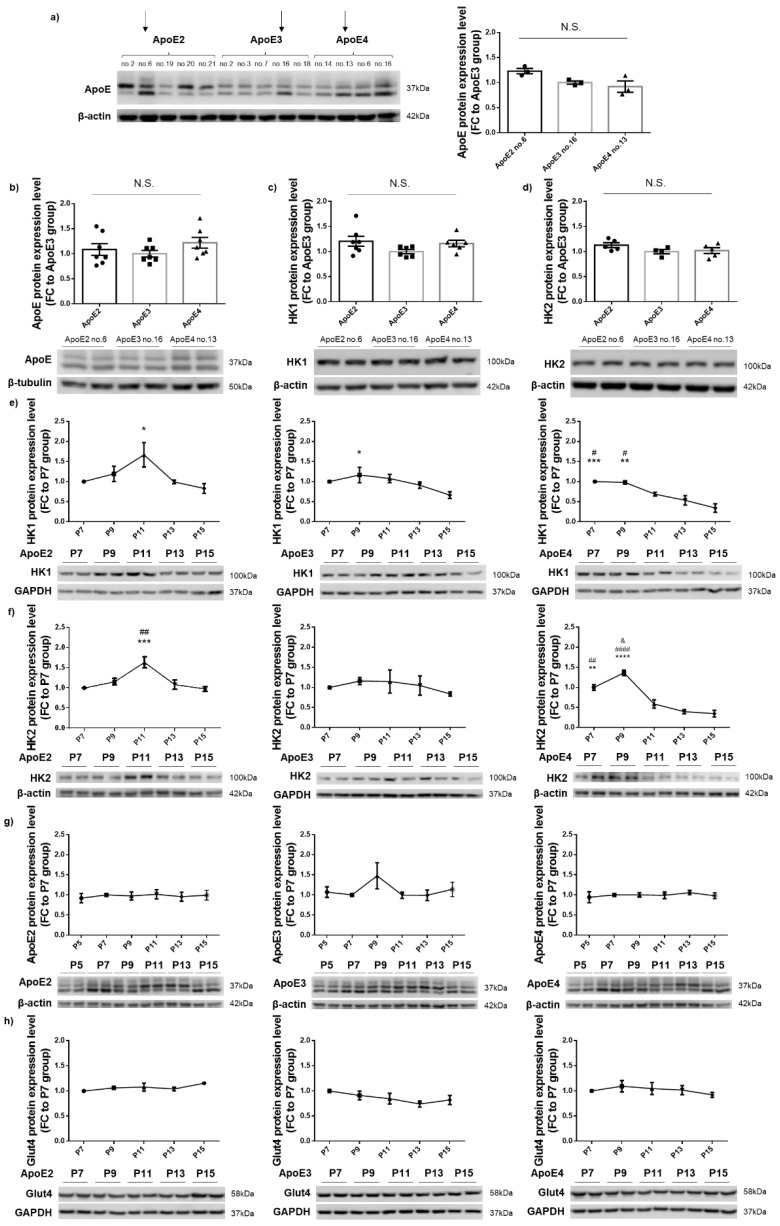
HK expression remained relatively stable in ApoE2- and ApoE3-expressing cells; in contrast, a gradual decrease with increasing passages occurred in ApoE4-expressing cells. (**a**) ApoE protein expression levels in human ApoE2-, ApoE3-, or ApoE4-expressing stable cell lines at P1. Results were normalized to the ApoE3 no. 16 line. n = 3 repeats/line. Three lines that showed similar ApoE levels (ApoE2 no. 6, ApoE3 no. 16, ApoE4 no. 13; indicated by arrows) were chosen and used in the following studies. (**b**–**d**) ApoE, HK1, and HK2 exhibited similar levels in the three chosen cell lines at P5. Results were normalized to the ApoE3 group. n = 4–7 repeats/line. (**e**–**h**) HK1 and HK2 protein levels progressively declined in ApoE4-expressing cells; however, ApoE and GLUT4 expression levels were unchanged in all three ApoE cells lines, from P7 to P15. Results were normalized to the P7 group. n = 2–6/group. * *p* < 0.05, ** *p* < 0.01, *** *p* < 0.001, **** *p* < 0.0001 vs. P15 group, # *p* < 0.05, ## *p* < 0.01, #### *p* < 0.0001 vs. P13 group, and & *p* < 0.05 vs. P7 group. N.S.: not significant.

N2a cells that stably expressed human ApoE2, ApoE3, or ApoE4 were generated as previously described [21]. A Western blot was performed to analyze the ApoE protein expression level in representative cell lines for each of the three isoforms at passage one (P1). Arrows indicate the cell lines of interest (ApoE2 no. 6, ApoE3 no. 16, and ApoE4 no. 13) that showed comparable levels of ApoE expression and were thus selected as the final set for the following studies (Figure 1a). ApoE protein levels in the three chosen cell lines at P5 remained consistent (Figure 1b). In addition, expression levels of both HK isoforms, HK1 and HK2, in these lines at P5 showed no significant differences (Figure 1c,d). Based on these results, we chose P5 as the starting point of the following time-course study designed to evaluate the interactive impact of ApoE and aging on HK expression. Cell lysates were harvested at every other passage, beginning at P7, and probed for HK1, HK2, ApoE, and glucose transporter type 4 (GLUT4). The expression of both HK1 and HK2 in ApoE4-expressing cells gradually decreased with increasing passage numbers starting at P11, and the reduction culminated at P15, the final passage studied. In contrast, the expression of HK1 and HK2 remained relatively unchanged from P7 to P15 in ApoE2- and ApoE3-expressing cells (Figure 1e,f). To determine whether HK changes could be caused by the fluctuation of cellular ApoE levels, human ApoE expression in the same cell lysates was also examined and was found to be unaltered across all the passages in all the three ApoE lines (Figure 1g). Similarly, GLUT4 expression was unchanged with increasing passages in all three lines (Figure 1h). Taken together, these results indicate that HK is differentially regulated by human ApoE isoforms, and it appears to be chronically and negatively affected by ApoE4. Moreover, the data indicate that the regulation of HK by ApoE isoforms is independent of protein levels of ApoE and GLUT4 in the cell.

### 3.2. ApoE2 Upregulated Whereas ApoE4 Downregulated HK, Which Occurred Concurrently with Downregulation of PFKP and PKM1 by ApoE2 and Upregulation by ApoE4

**Figure 2:**
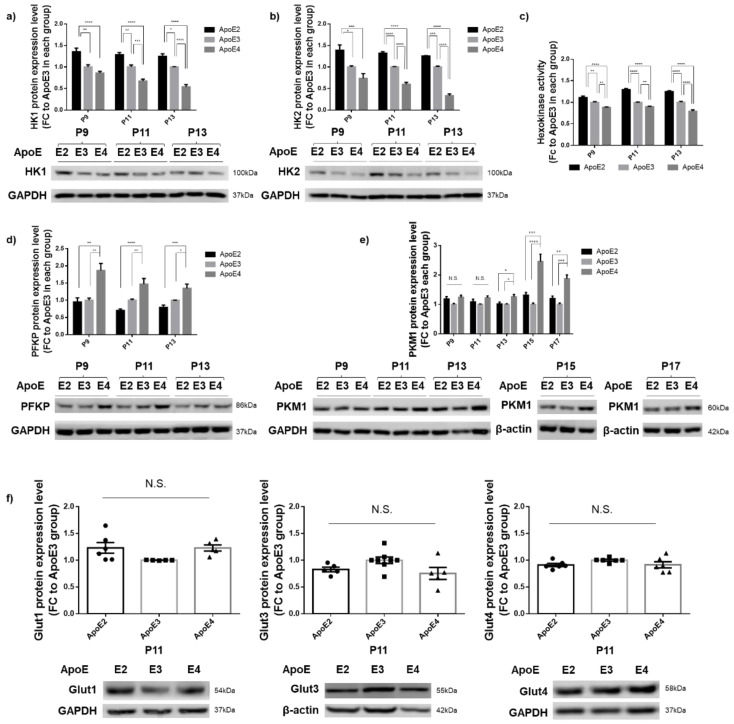
HK was upregulated in ApoE2-expressing cells and downregulated in ApoE4-expressing cells, which occurred concurrently with downregulation of PFKP and PKM1 by ApoE2 and upregulation by ApoE4. (**a**–**c**) Protein levels of both isoforms and activity of HK were upregulated in ApoE2-expressing cells and downregulated in ApoE4-expressing cells at all three passages, P9, P11, and P13. n = 5–9/group. (**d**,**e**) PFKP and PKM1 protein levels were significantly downregulated in ApoE4-expressing cells compared to ApoE2- and ApoE3-expressing cells. n = 4–7/group. (**f**) GLUT1, GLUT3, and GLUT4 protein levels showed no significant differences among the three ApoE cell lines at P11. n = 5–8/group. Results were normalized to the ApoE3 group in each passage. * *p* < 0.05, ** *p* < 0.01, *** *p* < 0.001, **** *p* < 0.0001. N.S.: not significant.

Following up on the observation that a significant decrease in HK protein expression started at P11 in ApoE4-expressing cells, we next analyzed the expression and activity of HK in the three ApoE cell lines at P9, P11, and P13. At all three passages, compared to ApoE3-expressing cells, ApoE2-expressing cells exhibited a higher expression of both HK1 and HK2, whereas ApoE4-expressing cells showed a significantly lower expression (Figure 2a,b). Consistent with the expression data, ApoE2-expressing cells exhibited a higher level of HK enzymatic activity, whereas ApoE4-expressing cells demonstrated decreased activity when compared to ApoE3 cells (Figure 2c). Apart from HK, phosphofructokinase (PFK) and pyruvate kinase (PK) also serve as the key rate-limiting enzymes involved in glycolysis. PFK is subject to allosteric regulation by the energy state of the cell and is stimulated by decreases in the ATP/AMP ratio (i.e., low energy state) [27]. The platelet isoform of PFK (PFKP) is the predominant form expressed in the brain [28,29]. PK, with the PKM1 isoform as the primary variant expressed in neurons, shows the sigmoidal kinetics in the allosteric inactivation by increased ATP and alanine [30,31]. Analyses of PFKP and PKM1 expression in the three ApoE cell lines showed that PFKP levels were significantly increased in ApoE4-expressing cells and decreased in ApoE2-expressing cells at all three passages, P9, P11, and P13 (Figure 2d). PKM1 exhibited similar changes—increases in ApoE4 cells and decreases in ApoE2 cells—although there was a slight delay starting at P13, and the changes culminated at P15–P17 (Figure 2e). Significant upregulation of both PFKP and PKM1 reflected the abnormally low energy state in the ApoE4-expressing cells, likely caused by downregulation of HK. Glucose transporters exist in several isoforms, including GLUT1–GLUT4, and GLUT1 and GLUT3 are found to be downregulated in AD brains [32]. In our analyses, all three GLUTs highly expressed in the brain, GLUT1, GLUT3, and GLUT4, showed no differences among the three ApoE cell lines at P11 (Figure 2f). In summary, these results indicate that ApoE2 upregulated whereas ApoE4 downregulated HK, which occurred concurrently with downregulation of PFKP and PKM1 by ApoE2 and upregulation by ApoE4. However, GLUTs are not affected by ApoE isoforms.

### 3.3. ApoE2-Expressing Cells Exhibited Enhanced Glycolytic Activity When Compared to ApoE4-Expressing Cells, Which Correlated with the General State of Cellular Health

**Figure 3:**
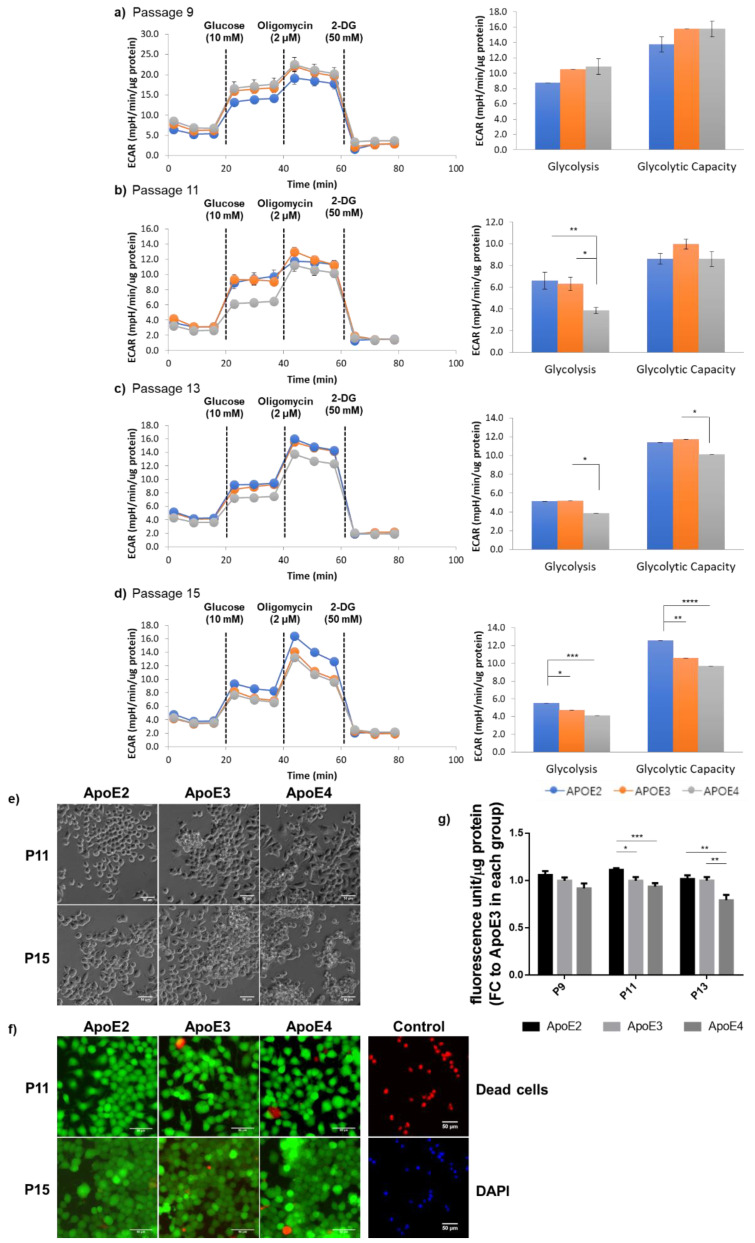
ApoE2- and ApoE4-expressing cells exhibited age-dependent divergent glycolytic profiles, which paralleled with changes in the general state of cellular health. (**a**–**d**) Glycolytic activity of ApoE-expressing cells at P9, P11, P13, and P15 was analyzed using a Seahorse XF96 extracellular flux analyzer. The ECAR readout was normalized by the total protein content in the sample in each well. n = 10–16/group. (**e**) Representative phase contrast images were obtained on the fourth day after cell seeding at indicated passages. (**f**) The LIVE/DEAD (green/red) cell staining was conducted and showed no significant cell death in any of the cell lines at up to P15. Image regions displayed had similar cell densities at about 80% confluency. Scale bars, 50 μm. (**g**) Metabolic activity of ApoE-expressing cells was analyzed using a Resazurin-based PrestoBlue assay in cells seeded on 96-well plates for 4 days and normalized by the total protein content in the sample in each well. n = 12–18/group. Results were normalized to the ApoE3 group in each passage. * *p* < 0.05, ** *p* < 0.01, *** *p* < 0.001, **** *p* < 0.0001.

Because HK is the first rate-limiting enzyme functioning as the “pacemaker” in glycolysis, glycolytic phenotypes in the three ApoE cell lines were then analyzed using a Seahorse glycolysis stress test kit. Starting at P11, the basal glycolytic rate exhibited the lowest in ApoE4-expressing cells. At P15, when cells reached a more advanced age, a greater decrease in the maximum glycolytic capacity induced by oligomycin occurred in ApoE4-expressing cells; in contrast, ApoE2-expressing cells exhibited a significantly higher readout than both ApoE3- and ApoE4-expressing cells (Figure 3a–d). These data indicate that ApoE2-expressing cells harnessed the most robust glycolytic activity, whereas ApoE4-expressing cells possessed a deficient phenotype that became increasingly severe in aged cells. After having observed the distinctive glycolytic profiles exhibited in the three ApoE cell lines, we next investigated whether these differences influenced the general health of the cells. Phase-contrast images revealed that ApoE2 cells presented with an evidently healthier appearance, whereas ApoE4 cells tended to aggregate into clumps, indicative of cells undergoing deteriorating changes. In line with the temporal development in the glycolytic changes, aggregation of ApoE4 cells started at P11 and worsened at P15 (Figure 3e). Moreover, consistent with the cell appearance, ApoE4 cells exhibited a similar time-dependent decline in the overall cellular metabolism assessed by a Resazurin-based assay that measures the reducing power of viable cells (Figure 3g). Despite the deterioration of general health, as clearly indicated by both cellular appearance and metabolic activity, a LIVE/DEAD cell staining assay did not find significant cell death in any of the three ApoE lines at up to P15 (Figure 3f). Therefore, it is possible that the cell aggregation observed in ApoE4 cultures could be caused by compromises to cell membranes that promoted cell clumps, and this detrimental process could accumulate into and culminate in intracellular damages, ultimately leading to cell death. These results, together with the data presented in Figure 2, indicate a strong and positive correlation between the cell’s glycolytic strength and the cell’s overall health status.

### 3.4. Differential Regulation of HK and Glycolytic Activity by ApoE Isoforms Was Also Observed in Differentiated Neuron-like Cells

**Figure 4:**
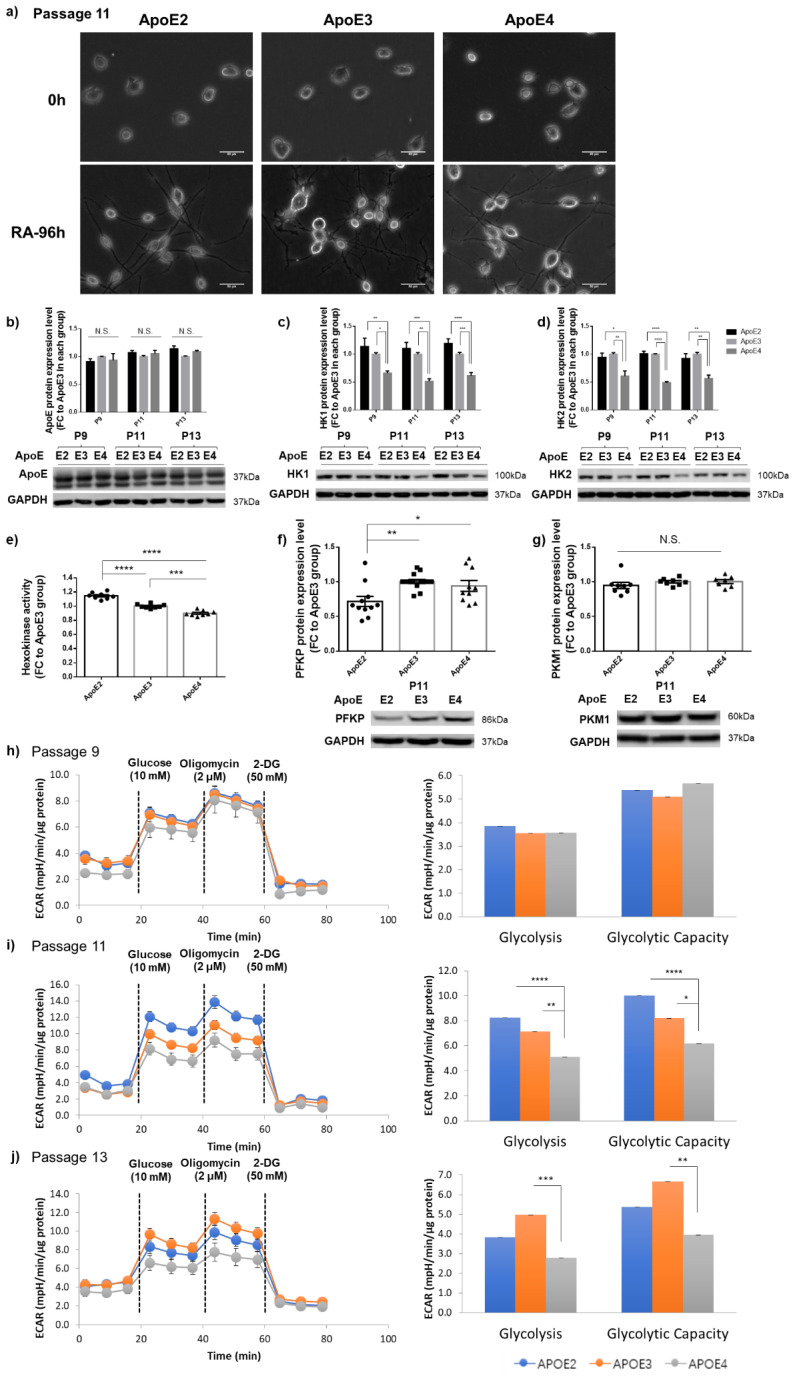
HK and glycolytic activity were differentially regulated in RA-induced human ApoE-expressing neuron-like cells. (**a**) Representative phase contrast images obtained from differentiated P11 cells demonstrated cellular processes consistent with neuron-like morphologies. Scale bars, 50 μm. (**b**) ApoE remained at similar levels among the three ApoE isoforms at P9, P11, and P13. n = 3–6/group. (**c**,**d**) Protein levels of both HK1 and Hk2 were elevated in ApoE2-expressing neuronal cells and reduced in ApoE4-expressing cells at P9, P11, and P13. n = 3–7/group. (**e**) Hexokinase activity was upregulated in ApoE2-expresing neuronal cells and downregulated in ApoE4-expressing neuronal cells. n = 8/group. (**f**,**g**) PFKP protein levels were significantly lower in ApoE2-expressing neuronal cells than in ApoE3 and ApoE4 cells at P11. n = 7–14/group. (**h**–**j**) ApoE4-expressing neuronal cells exhibited the lowest level on both the basal glycolytic rate and the maximum glycolytic capacity when compared to ApoE2 and ApoE3 neuronal cells at P11 and P13. n = 9–18/group. Results were normalized to the ApoE3 group in each passage. * *p* < 0.05, ** *p* < 0.01, *** *p* < 0.001, **** *p* < 0.0001. N.S.: not significant.

To study ApoE effects on neurons, we differentiated N2a-hApoE cells at P9, P11, and P13 by a 96 h treatment with retinoic acid (RA). Phase-contrast images demonstrated elaboration of cellular processes corresponding to the development of neuron-like morphologies, confirming the neuronal phenotype of RA-induced differentiated human ApoE-expressing cells (Figure 4a). Consistent with the data presented above, ApoE levels were no different among the three isoforms; however, expression of both HK1 and HK2, and HK activity, were upregulated in ApoE2-expressing and downregulated in ApoE4-expressing neuronal cells compared to ApoE3 neuronal cells (Figure 4b–e). Similarly, PFKP levels were considerably increased in ApoE4-expressing neuronal cells, pointing to the low energy state in those cells (Figure 4f). Moreover, consistent with the data generated in non-differentiated cells, PKM1 remained at similar levels among the three groups at P11 (Figure 4g); we speculated that significant differences could emerge when cells reached an advanced age. A Seahorse glycolysis stress test was further conducted. As predicted, at P11 and P13, 96 h RA-induced differentiated ApoE4-expressing neuronal cells exhibited the lowest level on both the basal glycolytic rate and the maximum glycolytic capacity compared to ApoE3 and ApoE4 neuronal cells (Figure 4h–j). In summary, these results demonstrate that HK and glycolytic metabolism are differentially regulated in human ApoE-expressing neuron-like cells in an age-dependent manner, further confirming our earlier findings in postmitotic neurons.

### 3.5. Introduction of ApoE2 Ameliorated HK Deficits and Further Improved Glycolytic Metabolism in ApoE4-Expressing Cells

**Figure 5:**
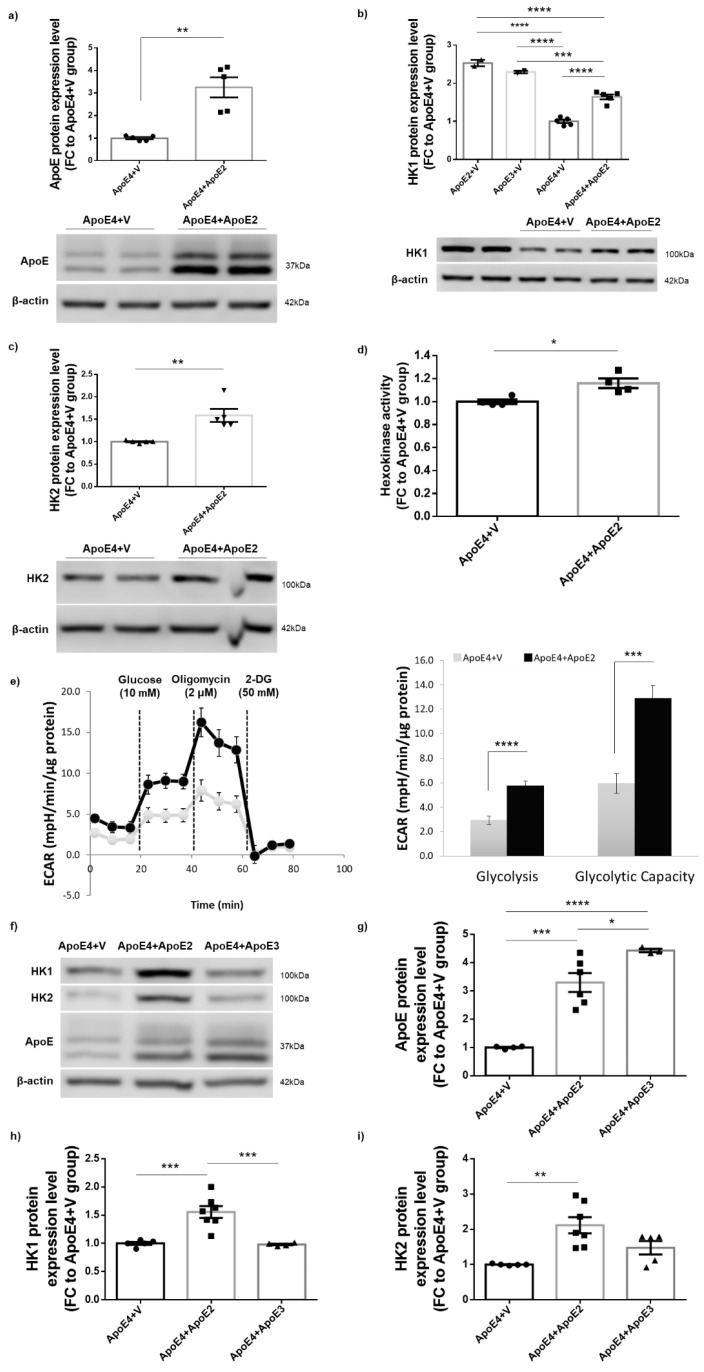
Expression of ApoE2 ameliorated HK deficits and improved glycolytic metabolism in ApoE4-expressing cells. (**a**) N2a-hApoE4 stable cells were transfected with human ApoE2 plasmids and maintained for 48–72 h. Cell lysates were harvested and analyzed for ApoE2 protein expression to confirm the transfection efficiency. n = 5/group. (**b**–**d**) Protein levels of both HK1 and HK2, and HK activity, were significantly higher in ApoE4 cells transfected with ApoE2 (ApoE4 + ApoE2) compared to ApoE4 cells transfected with empty vector (ApoE4 + V). n = 2–5/group. (**e**) Glycolytic readouts from a Seahorse stress test were markedly increased in ApoE4 + ApoE2 cells compared to ApoE4 + V cells. n = 8/group. (**f**–**i**) N2a-hApoE4 stable cells were transfected with human ApoE2 or ApoE3 plasmids and maintained for 48–72 h. Expression of ApoE2 but not ApoE3 increased HK expression in ApoE4-expressing cells. n = 3–7/group. Results were normalized to the ApoE4 + V group. * *p* < 0.05, ** *p* < 0.01, *** *p* < 0.001, **** *p* < 0.0001.

Our findings of strong correlations between cellular glycolytic status and general wellness led us to hypothesize that introduction of the ApoE2 protein into ApoE4-expressing cells would reverse or ameliorate ApoE4-associated HK deficit and glycolytic hypometabolism. To test this hypothesis, N2a-hApoE4 stable cells were transfected with mammalian expression vector encoding human ApoE2 (ApoE4 + ApoE2) or empty vector (ApoE4 + V). 48–72 h after transfection, cells were analyzed for HK protein expression, HK enzymatic activity, and cellular responses to glycolytic stresses. Transfection induced a nearly three-fold higher expression of ApoE2 protein in ApoE4-expressing cells than in ApoE + V cells (Figure 5a). Both HK1 and HK2 protein levels, as well as HK activity, were significantly increased in ApoE4 + ApoE2 cells (Figure 5b–d). In a Seahorse glycolytic stress test, ApoE4 + ApoE2 cells exhibited drastically increased ECAR readout for both the basal glycolytic rate and the maximum glycolytic capacity when compared to ApoE4 + V cells. However, expression of ApoE3 in ApoE4 cells did not exert a rescue effect, indicating the unique neuroprotective property of ApoE2 (Figure 5f–i). Collectively, these data indicate that introduction of ApoE2 could rescue ApoE4-associated HK expression deficits and improve glycolytic function.

### 3.6. PI3K/Akt Signaling Activity Appeared to Be Responsible for ApoE2-Mediated Upregulation of HK2 but Not of HK1 Expression

**Figure 6:**
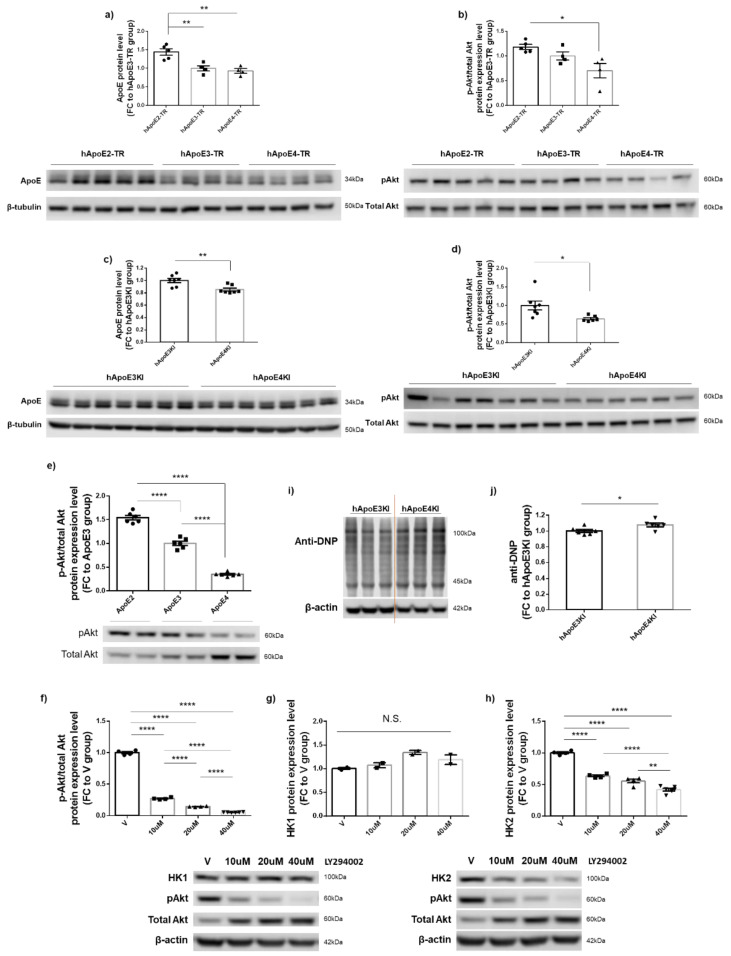
ApoE-dependent PI3K/Akt signaling activity modulated HK2 but not HK1 protein expression. (**a**–**d**) ApoE and pAkt/tAkt ratio were upregulated in ApoE2 and ApoE3 mouse cortex and downregulated in ApoE4 mice. Human ApoE-TR mice were obtained from Taconic; human ApoEKI mice were obtained from JAX Laboratories; both sets of mice were at ages of 6–7 months. Results were normalized to the ApoE3 mice group. n = 4–7 mice/group. (**e**) Consistent with the mouse data, pAkt/tAkt ratio was significantly upregulated in N2a-hApoE2 stable cells and downregulated in N2a-hApoE4 cells at P11. Results were normalized to N2a-hApoE3 cells. n = 6/group. (**f**–**h**) N2a-hApoE2 cells at P11 were treated with serial concentrations of the PI3 inhibitor, LY294002, for 24 h; cell lysates were analyzed for changes in pAkt/tAkt expression ratio and protein levels of both HK1 and HK2. LY294002 induced a dose-dependent decrease in pAkt/tAkt ratio and HK2 expression; however, HK1 expression was unaltered. Results were normalized to the vehicle-treated group. n = 2–6/group. (**i**,**j**) Total protein carbonylation levels were slightly increased in 6–7-month-old ApoE4 mouse cortex compared to ApoE3 mice. n = 6 mice/group. * *p* < 0.05, ** *p* < 0.01, **** *p* < 0.0001. N.S.: not significant.

PI3K/Akt signaling cascades play important roles in cell metabolism, growth, proliferation, and survival, and have been shown to be differentially affected by ApoE isoforms [33,34]. Additionally, PI3K/Akt-dependent HK2 expression has been shown to contribute to cellular bioenergetics and survival [35]. To understand the regulatory mechanism leading to ApoE-mediated effects on HK expression, we next examined the PI3K/Akt activity in two different human ApoE-expressing mouse models—hApoE-TR mice obtained from Taconic and hApoEKI mice obtained from JAX Laboratories—and in N2a-hApoE stable cell lines at P11. Consistent with the previous report [36], we detected isoform-dependent differences in ApoE protein expression in the brains of both ApoE mouse models at 6–7 months of age (Figure 6a,c). The ratio of phosphorylated Akt/total Akt (pAkt/tAkt) protein expression levels also showed isoform-dependent differences, upregulated in ApoE2 mouse brains, and downregulated in ApoE4 mouse brains (Figure 6b,d). Consistent with the mouse data, the ratio of pAkt/tAkt expression levels was the highest in N2a-hApoE2 stable cells and the lowest in N2a-hApoE4 cells (Figure 6e). To further examine the role of the PI3K/Akt singling activity in the mediation of ApoE-dependent HK expression, we treated ApoE2 stable cells at P11 with the PI3K inhibitor, LY294002. Exposure of cells to LY294002 for 24 h resulted in a dose-dependent decrease in the pAkt/tAkt ratio along with HK2 but not HK1 expression (Figure 6f–h). We speculated that the increases in total Akt expression could be a compensatory response of cells to pAkt deficits. Considering the well-established role of PI3K/Akt in oxidative stress, we measured total protein carbonylation in cortical tissue collected from 6–7-month-old ApoE3KI and ApoE4KI mice of both sexes. Protein carbonylation is the most common form of protein oxidation, which can be induced directly by ROS or indirectly by secondary byproducts of oxidative stress [37]. As expected, we observed a slightly increased level of carbonylated proteins in ApoE4 brains compared to that in ApoE3 brains (Figure 6i–j). In summary, these results confirm that ApoE isoforms differentially modulate PI3K/Akt signaling cascades; however, PI3K/Akt activity appears to be only responsible for ApoE2-mediated upregulation of HK2 but not of HK1.

## 4. Discussion

**Figure 7:**
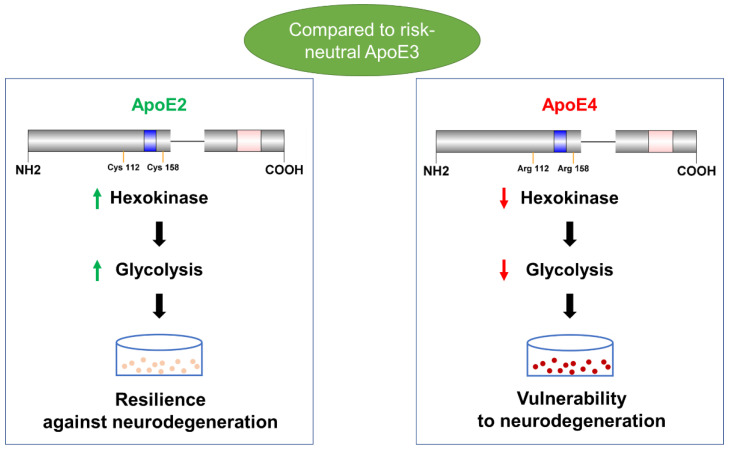
In conclusion, our study demonstrates that human ApoE2 isoform upregulates, whereas ApoE4 variant downregulates, neuronal HK and glycolytic activity. We further show that these divergent ApoE2 and ApoE4 effects on glycolysis significantly impact neuronal health and aging. These findings imply that neuronal glycolytic phenotypes may serve as a key contributor to ApoE-mediated brain resilience or vulnerability to AD. Blue: receptor binding region. Pink: lipid binding region.

ApoE ε4 increases LOAD risk, whereas ApoE ε2 reduces the risk [38]. ApoE4 carriers showed bioenergetic deficits preceding the onset of AD symptoms [39,40]. Prior results from our lab revealed that ApoE isoforms influenced cerebral glucose cytosolic metabolism [20]. In this follow-up study, we used human ApoE stable cell lines and neuronal models to further show that ApoE2- and ApoE4-dependent differences in glycolytic metabolism—ApoE2 upregulation and ApoE4 downregulation—impact neuronal health and aging, and that HK may mediate these effects (Figure 7). Moreover, we found that introduction of the ApoE2 protein can mitigate the ApoE4-associated reduction in HK expression and glycolysis flux. Collectively, our findings may help explain ApoE2-mediated neuroprotection and suggest potential AD-relevant therapeutic approaches.

Recent studies indicate relationships exist between glycolytic activity, AD pathology, and AD symptoms [12,13]. This is, perhaps, not surprising because glycolysis contributes to increased ATP synthesis driven by synaptic activity [41]. Proper functions of ion pumps, such as the V-type H^+^-ATPase (V-ATPase) and Na^+^/K^+^-ATPase, have been linked to membrane-bound glycolytic enzymes, indicating the critical role of glycolysis-derived ATP as an important source of energy in ion transport, which is vital for neurotransmitter release and synaptic transmission [42]. Specifically, the dependence of V-ATPase on glycolysis has been widely indicated by its close interaction with PFK, aldolase, and HK [15]. HK inhibition causes V-ATPase disassembly and functional loss in a PI3K-dependent manner [43,44]. We previously demonstrated that V-ATPase subunit expression is upregulated in ApoE2 brains [45]. Data presented herein confirmed that the ApoE2 genotype retains the most robust glycolytic profile during neuronal aging. Therefore, the enhanced glycolysis and the ensuing synaptic strength could potentially serve as a significant mechanism conferring cognitive resilience to ApoE2 carriers against aging-associated risks for LOAD.

HK is the first key enzyme in glycolysis responsible for phosphorylating glucose to glucose-6-phosphate (G6P). HK activity is found to be decreased in the brains, skin-cultured fibroblasts, and leukocytes of AD patients [46,47]. Our findings indicate that ApoE4 progressively impairs HK and that the impact of ApoE4 culminates in aged cells, resulting in a reduction in glycolytic activity. PFK, the second irreversible glycolytic enzyme, is subject to allosteric regulation dependent on cell energy status [48,49]. In our study, PFK upregulation occurred concurrently with HK downregulation in ApoE4 cells, which could reflect a compensatory reversal of the glycolytic deficit caused by HK hypoactivity in the cell. PK, the third major glycolytic enzyme, is found increased in cells exposed to AD plasma [50] and in AD brains [49]. In our study, like the changes to PFK, an increase in PK could be a cellular attempt to stimulate glycolytic metabolism. However, despite the elevated expression of PFK and PK, ApoE4 cells remained low on glycolytic activity, indicating the limited rescuing capability of PFK and PK. Moreover, levels of all three GLUTs—GLUT1, GLUT3, and GLUT4—were no different among the three ApoE cell lines, excluding a possible contribution of glucose availability to the glycolytic differences observed in these cells. Together, these observations highlight the role of HK in shaping the glycolytic phenotype associated with the three ApoE variants.

It should be noted that ApoE-mediated effects on glycolysis appear to be cell-type dependent. In line with our results, Zhao et al. demonstrated that, in *Apoe^−/−^* primary neurons, ApoE4 failed to induce insulin-stimulated glycolysis compared to ApoE3 [51]. Studies by Orr et al. showed that under stressed conditions, ApoE4-expressing neurons exhibited 50% less glycolytic reserve capacity [52]. In contrast to these findings, an opposite direction involving an increase in glycolysis by ApoE4 was found in astrocytes [53,54]. A higher dependence and capacity for using glucose by ApoE4 astrocytes was indicated by their enhanced basal respiration and glycolytic rate [55]. Serving in a supportive role, astrocytes possess a remarkably divergent metabolic phenotype from neurons. For example, in the proposed astrocyte-neuron lactate shuttle (ANLS), astrocytes function to provide lactate generated in glycolysis as an energy substrate to neurons [56]. In AD and ApoE4 carriers, increased astrocytic glycolysis potentially acts as a compensation for neuronal energy deficit. Regarding the regulation of glycolysis in microglia, ApoE4-expressing iMGLs (human microglia-like cells) were found to exhibit reduced glycolysis flux under normal conditions, whereas proinflammatory stimulus upregulated it [57]. Moreover, research led by Fang et al. showed that compared with ApoE3, the glycolytic function of ApoE4 astrocytes was not significantly altered in one-month cultures, but exhibited a marked decrease in two-month cultures, suggesting a similar time-dependent impact of ApoE on astrocytic glycolysis [58]. Our findings help fill the gap in understanding the role of ApoE isoforms in neuronal glycolysis and demonstrate how it affects neuronal health in the aging process.

The growing literature accumulated over the last two decades has revealed an inverse association between cancer and AD. Individuals with a history of cancer are less likely to develop AD, whereas people diagnosed with AD exhibit a reduced risk of having cancer, independent of ethnic groups or environmental factors [59,60,61,62,63,64]. The origins of this inverse relationship are unknown, although recent studies suggest that the divergent trajectories of cellular bioenergetics involved in these two distinct diseases may be a contributor. The Warburg effect, the preferential use of glycolysis over oxidative phosphorylation (OXPHOS), is a hallmark of cancer, a disease characterized by a high rate of ATP synthesis to promote cellular division. Conversely, an inverse-Warburg effect involving an increased OXPHOS activity is increasingly recognized in the early stages of neurodegenerative diseases [65,66,67,68,69]. It can be reasonably speculated that neuronal hypometabolism associated with early AD, possibly due to an impaired glycolysis, leads to upregulation of lactate uptake through ANLS, thus giving rise to a compensatory increase in OXPHOS along with reactive oxygen species (ROS). Thereby, over time, accumulative lactate release by astrocytes in the brain can be detrimental and contribute to AD pathogenesis [70,71]. Based on our results, we hypothesize that ApoE isoforms differentially impact AD via an inverse Warburg mechanism. The ApoE2 genotype increases neuronal glycolysis, which translates into a more Warburgian phenotype and less ROS. In contrast, ApoE4 downregulates neuronal glycolysis, which promotes an inverse-Warburg environment that increases neuronal vulnerability to oxidative stress and neurodegeneration.

Apart from bioenergetics, the Warburg effect is also critical in supporting biosynthesis via the pentose phosphate pathway (PPP) and the hexosamine biosynthetic pathway (HBP), two major metabolic pathways diverted from G6P. PPP, which maintains the NADPH level in response to the synaptic stimulation and increased oxidative stress, has been shown to be greatly impaired in AD [72,73,74]. In the HBP, fructose-6-phosphate (F6P) derived from G6P is first converted to glucosamine-6-phosphate (GlcN6P). GlcN6P is then subjected to several steps of enzymatic reactions yielding uridine diphosphate N-acetylglucosamine (UDP-GlcNAc), the end product of the HBP. UDP-GlcNAc serves as the donor in protein post-translational modification (PTM) with O-linked β-N-acetylglucosamine (O-GlcNAc), a PTM process referred to as O-GlcNAcylation. O-GlcNAc is found significantly concentrated at neuronal synapses, and elevation of O-GlcNAcylation has been suggested as a promising strategy for AD [15,75]. UDP-GlcNAc can also be metabolized to N-acetyl-D-mannosamine (ManNAc) and, subsequently, to ManNAc-6-P as the first steps in the biosynthesis of sialic acid, the sugar donor used in sialylation, another very important PTM that plays critical roles in the central nervous system [76]. Indeed, our lab has recently first reported that ApoE isoforms in human brains undergo varying degrees of sialic acid modifications, with ApoE2 most extensively sialylated, which ameliorates Aβ fibrillation, whereas ApoE4 is least sialylated [77]. Further work is needed to determine whether these PTM differences may result from differences in HK and glycolytic profiles associated with the three ApoE isoforms. We would like to point out that because the linker region of the plasmids we used in this study introduced an artificial N-glycosylation site, the exhibited PTM (Figure 1) does not precisely represent the real glycosylation status of ApoE in those cells.

Recent studies show that glycolysis may also be involved in the regulation of mitochondrial function. HK is found to bind to the outer membrane of mitochondria by interacting with the voltage-dependent anion channel (VDAC). Mitochondrial bound hexokinase (mtHK) has long been recognized for its role in increasing the resistance of cancer cells to mitochondria-initiated apoptotic cell death [35,78]. Evidence indicates that mtHK suppresses cytochrome c release, possibly by interfering with the binding of Bax with VDAC and through further inhibition of the mitochondrial permeability transition pore [79,80,81,82]. Moreover, mtHK has been shown to be involved in the regulation of ADP recycling, contributing to a high efficiency of ATP production, energy preservation, and reduced ROS generation [83]. Recently, Chen et al. reported the colocalization of ApoE and VDAC in H9c2 cells and found that ApoE4 induced greater mitochondrial swelling compared to ApoE3 both in vitro and in vivo; however, the underlying mechanism is unknown [84]. Here, our data demonstrate that the ApoE4 genotype is associated with impaired HK expression and activity, which could potentially weaken the above mentioned anti-apoptotic and antioxidant mechanisms leading to declining cellular health. Notably, cell death was not observed within the passages studied. This observation is, in fact, in line with the trajectory of AD development in humans. AD is a unique disease in that it starts with a very long prodromal phase that can last up to 10–20 years, and during this period, various molecular changes can take place that culminate in neuronal death and the onset of clinical symptoms.

Gene delivery of ApoE2 in AD mouse models has been shown to be beneficial for alleviating brain amyloid pathology and neurotoxicity [85,86]. In our study, we found that introducing the ApoE2 protein by transient transfection in ApoE4-expressing cells can significantly improve HK expression and glycolytic activity, indicating a potential mechanism that underlies the neuroprotective effect by ApoE2. However, we did not observe a dose-dependent rescue effect of ApoE2 and more importantly, ApoE3 failed to induce a significant change in ApoE4 cells. These results confirmed a unique neuroprotective role of ApoE2 compared to ApoE3. With respect to the regulatory mechanism, according to our initial data, in 6–7-month-old human ApoE4-KI mouse brains, PI3K/Akt activity is downregulated with a concurrent reduction in HK and an elevation in total protein carbonylation, a marker of the overall level of oxidative stress. However, PI3K/Akt signaling appears to be only partially responsible for ApoE-mediated HK regulation. Several recent studies demonstrate that ApoE acts as a direct transcription factor in the expression of genes related to aging and AD [87,88]. Further work is needed to fully understand the underlying mechanisms governing the regulation of HK by ApoE.

## 5. Conclusions

The present study highlights the potentially crucial role of HK-driven glycolytic phenotypes in conferring neuronal resilience or vulnerability associated with ApoE genetic variants to AD. Our findings lend compelling support to the recently proposed inverse-Warburg origin of AD and present a novel and exciting opportunity in the prevention and treatment of AD [16,66,67,89].

## Data Availability

The data that support the findings of this study are available from the corresponding author upon reasonable request.

## References

[1] Mahley R.W., Huang Y. (2012). Apolipoprotein e sets the stage: Response to injury triggers neuropathology. Neuron.

[2] Mahley R.W., Rall S.C. (2000). Apolipoprotein E: Far more than a lipid transport protein. Annu. Rev. Genom. Hum. Genet..

[3] Liu C.-C., Liu C.-C., Kanekiyo T., Xu H., Bu G. (2013). Apolipoprotein E and Alzheimer disease: Risk, mechanisms and therapy. Nat Rev. Neurol..

[4] Alzheimer’s Association (2021). 2020 Alzheimer’s Disease Facts and Figures. Alzheimers Dement..

[5] Morris C.M., Benjamin R., Leake A., McArthur F.K., Candy J.M., Ince P.G., Torvik A., Bjertness E., Edwardson J.A. (1995). Effect of apolipoprotein E genotype on Alzheimer’s disease neuropathology in a cohort of elderly Norwegians. Neurosci. Lett..

[6] Nagy Z., Esiri M.M., Jobst K.A., Johnston C., Litchfield S., Sim E., Smith A.D. (1995). Influence of the apolipoprotein E genotype on amyloid deposition and neurofibrillary tangle formation in Alzheimer’s disease. Neuroscience.

[7] Wilson R.S., Bienias J.L., Berry-Kravis E., Evans D.A., Bennett D.A. (2002). The apolipoprotein E epsilon 2 allele and decline in episodic memory. J. Neurol. Neurosurg. Psychiatry.

[8] Mosconi L., De Santi S., Li J., Tsui W.H., Li Y., Boppana M., Laska E., Rusinek H., de Leon M.J. (2008). Hippocampal hypometabolism predicts cognitive decline from normal aging. Neurobiol. Aging.

[9] Swerdlow R.H., Burns J.M., Khan S.M. (2014). The Alzheimer’s disease mitochondrial cascade hypothesis: Progress and perspectives. Biochim. Biophys. Acta.

[10] Swerdlow R., Marcus D.L., Landman J., Kooby D., Frey W., Freedman M.L. (1994). Brain glucose metabolism in Alzheimer’s disease. Am. J. Med. Sci..

[11] Johnson L.A., Söderbom G., Esterline R., Oscarsson J., Mattson M.P. (2020). Chapter Five—APOE and metabolic dysfunction in Alzheimer’s disease. International Review of Neurobiology.

[12] An Y., Varma V.R., Varma S., Casanova R., Dammer E., Pletnikova O., Chia C.W., Egan J.M., Ferrucci L., Troncoso J. (2018). Evidence for brain glucose dysregulation in Alzheimer’s disease. Alzheimers Dement. J. Alzheimers Assoc..

[13] Vlassenko A.G., Gordon B.A., Goyal M.S., Su Y., Blazey T.M., Durbin T.J., Couture L.E., Christensen J.J., Jafri H., Morris J.C. (2018). Aerobic glycolysis and tau deposition in preclinical Alzheimer’s disease. Neurobiol. Aging.

[14] Theurey P., Connolly N.M.C., Fortunati I., Basso E., Lauwen S., Ferrante C., Moreira Pinho C., Joselin A., Gioran A., Bano D. (2019). Systems biology identifies preserved integrity but impaired metabolism of mitochondria due to a glycolytic defect in Alzheimer’s disease neurons. Aging Cell.

[15] Zhang X., Alshakhshir N., Zhao L. (2021). Glycolytic Metabolism, Brain Resilience, and Alzheimer’s Disease. Front. Neurosci..

[16] Newington J.T., Pitts A., Chien A., Arseneault R., Schubert D., Cumming R.C. (2011). Amyloid beta resistance in nerve cell lines is mediated by the Warburg effect. PLoS ONE.

[17] Mosconi L., De Santi S., Brys M., Tsui W.H., Pirraglia E., Glodzik-Sobanska L., Rich K.E., Switalski R., Mehta P.D., Pratico D. (2008). Hypometabolism and altered cerebrospinal fluid markers in normal apolipoprotein E E4 carriers with subjective memory complaints. Biol. Psychiatry.

[18] Reiman E.M., Caselli R.J., Yun L.S., Chen K., Bandy D., Minoshima S., Thibodeau S.N., Osborne D. (1996). Preclinical evidence of Alzheimer’s disease in persons homozygous for the epsilon 4 allele for apolipoprotein E. N. Engl. J. Med..

[19] Harris R.A., Lone A., Lim H., Martinez F., Frame A.K., Scholl T.J., Cumming R.C. (2019). Aerobic Glycolysis Is Required for Spatial Memory Acquisition But Not Memory Retrieval in Mice. Eneuro.

[20] Wu L., Zhang X., Zhao L. (2018). Human ApoE Isoforms Differentially Modulate Brain Glucose and Ketone Body Metabolism: Implications for Alzheimer’s Disease Risk Reduction and Early Intervention. J. Neurosci. Off. J. Soc. Neurosci..

[21] Wu L. (2017). Perturbed Brain Energy Metabolism in Alzheimer’s Disease and Diabetes. Ph.D. Thesis.

[22] Tremblay R.G., Sikorska M., Sandhu J.K., Lanthier P., Ribecco-Lutkiewicz M., Bani-Yaghoub M. (2010). Differentiation of mouse Neuro 2A cells into dopamine neurons. J. Neurosci. Methods.

[23] Wu P.Y., Lin Y.C., Chang C.L., Lu H.T., Chin C.H., Hsu T.T., Chu D., Sun S.H. (2009). Functional decreases in P2X7 receptors are associated with retinoic acid-induced neuronal differentiation of Neuro-2a neuroblastoma cells. Cell. Signal..

[24] Sullivan P.M., Mace B.E., Maeda N., Schmechel D.E. (2004). Marked regional differences of brain human apolipoprotein E expression in targeted replacement mice. Neuroscience.

[25] Jankowsky J.L., Zheng H. (2017). Practical considerations for choosing a mouse model of Alzheimer’s disease. Mol. Neurodegener..

[26] Ding F., Yao J., Rettberg J.R., Chen S., Brinton R.D. (2013). Early decline in glucose transport and metabolism precedes shift to ketogenic system in female aging and Alzheimer’s mouse brain: Implication for bioenergetic intervention. PLoS ONE.

[27] Poorman R.A., Randolph A., Kemp R.G., Heinrikson R.L. (1984). Evolution of phosphofructokinase—Gene duplication and creation of new effector sites. Nature.

[28] Fernandes P.M., Kinkead J., McNae I., Michels P.A.M., Walkinshaw M.D. (2020). Biochemical and transcript level differences between the three human phosphofructokinases show optimisation of each isoform for specific metabolic niches. Biochem. J..

[29] Agostini M., Romeo F., Inoue S., Niklison-Chirou M.V., Elia A.J., Dinsdale D., Morone N., Knight R.A., Mak T.W., Melino G. (2016). Metabolic reprogramming during neuronal differentiation. Cell Death Differ..

[30] Carbonell J., Marco R., Felíu J.E., Sols A. (1973). Pyruvate Kinase. Eur. J. Biochem..

[31] Magistretti P.J., Allaman I. (2015). A cellular perspective on brain energy metabolism and functional imaging. Neuron.

[32] Szablewski L. (2017). Glucose Transporters in Brain: In Health and in Alzheimer’s Disease. J. Alzheimers Dis. JAD.

[33] Huang Y.A., Zhou B., Nabet A.M., Wernig M., Südhof T.C. (2019). Differential Signaling Mediated by ApoE2, ApoE3, and ApoE4 in Human Neurons Parallels Alzheimer’s Disease Risk. J. Neurosci. Off. J. Soc. Neurosci..

[34] Laffont I., Takahashi M., Shibukawa Y., Honke K., Shuvaev V.V., Siest G., Visvikis S., Taniguchi N. (2002). Apolipoprotein E activates Akt pathway in neuro-2a in an isoform-specific manner. Biochem. Biophys. Res. Commun..

[35] Roberts D.J., Miyamoto S. (2015). Hexokinase II integrates energy metabolism and cellular protection: Akting on mitochondria and TORCing to autophagy. Cell Death Differ..

[36] Riddell D.R., Zhou H., Atchison K., Warwick H.K., Atkinson P.J., Jefferson J., Xu L., Aschmies S., Kirksey Y., Hu Y. (2008). Impact of apolipoprotein E (ApoE) polymorphism on brain ApoE levels. J. Neurosci. Off. J. Soc. Neurosci..

[37] Suzuki Y.J., Carini M., Butterfield D.A. (2010). Protein carbonylation. Antioxid. Redox. Signal..

[38] Li Z., Shue F., Zhao N., Shinohara M., Bu G. (2020). APOE2: Protective mechanism and therapeutic implications for Alzheimer’s disease. Mol. Neurodegener..

[39] Drzezga A., Riemenschneider M., Strassner B., Grimmer T., Peller M., Knoll A., Wagenpfeil S., Minoshima S., Schwaiger M., Kurz A. (2005). Cerebral glucose metabolism in patients with AD and different APOE Genotypes. Neurology.

[40] Mosconi L. (2005). Brain glucose metabolism in the early and specific diagnosis of Alzheimer’s disease. Eur. J. Nucl. Med. Mol. Imaging.

[41] Goyal M.S., Hawrylycz M., Miller J.A., Snyder A.Z., Raichle M.E. (2014). Aerobic glycolysis in the human brain is associated with development and neotenous gene expression. Cell Metab..

[42] Moriyama Y., Maeda M., Futai M. (1992). The role of V-ATPase in neuronal and endocrine systems. J. Exp. Biol..

[43] Kohio H.P., Adamson A.L. (2013). Glycolytic control of vacuolar-type ATPase activity: A mechanism to regulate influenza viral infection. Virology.

[44] Nakamura S. (2004). Glucose activates H+-ATPase in kidney epithelial cells. Am. J. Physiol.-Cell Physiol..

[45] Woody S.K., Zhou H., Ibrahimi S., Dong Y., Zhao L. (2016). Human ApoE ɛ2 Promotes Regulatory Mechanisms of Bioenergetic and Synaptic Function in Female Brain: A Focus on V-type H +-ATPase. J. Alzheimers Dis..

[46] Saraiva L.M., Seixas da Silva G.S., Galina A., da-Silva W.S., Klein W.L., Ferreira S.T., De Felice F.G. (2010). Amyloid-β triggers the release of neuronal hexokinase 1 from mitochondria. PLoS ONE.

[47] Sorbi S., Mortilla M., Piacentini S., Tonini S., Amaducci L. (1990). Altered hexokinase activity in skin cultured fibroblasts and leukocytes from Alzheimer’s disease patients. Neurosci. Lett..

[48] Bigl M., Bleyl A.-D., Zedlick D., Arendt T., Bigl V., Eschrich K. (1996). Changes of Activity and Isozyme Pattern of Phosphofructokinase in the Brains of Patients with Alzheimer’s Disease. J. Neurochem..

[49] Bigl M., Brückner M.K., Arendt T., Bigl V., Eschrich K. (1999). Activities of key glycolytic enzymes in the brains of patients with Alzheimer’s disease. J. Neural Transm..

[50] Jayasena T., Poljak A., Braidy N., Smythe G., Raftery M., Hill M., Brodaty H., Trollor J., Kochan N., Sachdev P. (2015). Upregulation of glycolytic enzymes, mitochondrial dysfunction and increased cytotoxicity in glial cells treated with Alzheimer’s disease plasma. PLoS ONE.

[51] Zhao N., Liu C.C., Van Ingelgom A.J., Martens Y.A., Linares C., Knight J.A., Painter M.M., Sullivan P.M., Bu G. (2017). Apolipoprotein E4 Impairs Neuronal Insulin Signaling by Trapping Insulin Receptor in the Endosomes. Neuron.

[52] Orr A.L., Kim C., Jimenez-Morales D., Newton B.W., Johnson J.R., Krogan N.J., Swaney D.L., Mahley R.W. (2019). Neuronal Apolipoprotein E4 Expression Results in Proteome-Wide Alterations and Compromises Bioenergetic Capacity by Disrupting Mitochondrial Function. J. Alzheimers Dis. JAD.

[53] Williams H.C., Farmer B.C., Piron M.A., Walsh A.E., Bruntz R.C., Gentry M.S., Sun R.C., Johnson L.A. (2020). APOE alters glucose flux through central carbon pathways in astrocytes. Neurobiol. Dis..

[54] Farmer B.C., Williams H.C., Devanney N.A., Piron M.A., Nation G.K., Carter D.J., Walsh A.E., Khanal R., Young L.E.A., Kluemper J.C. (2021). APOΕ4 lowers energy expenditure in females and impairs glucose oxidation by increasing flux through aerobic glycolysis. Mol. Neurodegener..

[55] Qi G., Mi Y., Shi X., Gu H., Brinton R.D., Yin F. (2021). ApoE4 Impairs Neuron-Astrocyte Coupling of Fatty Acid Metabolism. Cell Rep..

[56] Pellerin L., Magistretti P.J. (1994). Glutamate uptake into astrocytes stimulates aerobic glycolysis: A mechanism coupling neuronal activity to glucose utilization. Proc. Natl. Acad. Sci. USA.

[57] Konttinen H., Cabral-da-Silva M.E.C., Ohtonen S., Wojciechowski S., Shakirzyanova A., Caligola S., Giugno R., Ishchenko Y., Hernández D., Fazaludeen M.F. (2019). PSEN1ΔE9, APPswe, and APOE4 Confer Disparate Phenotypes in Human iPSC-Derived Microglia. Stem Cell Rep..

[58] Fang W., Xiao N., Zeng G., Bi D., Dai X., Mi X., Ye Q., Chen X., Zhang J. (2021). APOE4 genotype exacerbates the depression-like behavior of mice during aging through ATP decline. Transl. Psychiatry.

[59] Li J.-M., Liu C., Hu X., Cai Y., Ma C., Luo X.-G., Yan X.-X. (2014). Inverse correlation between Alzheimer’s disease and cancer: Implication for a strong impact of regenerative propensity on neurodegeneration?. BMC Neurol..

[60] Lanni C., Masi M., Racchi M., Govoni S. (2021). Cancer and Alzheimer’s disease inverse relationship: An age-associated diverging derailment of shared pathways. Mol. Psychiatry.

[61] (2013). Skin cancer—Protective effect against Alzheimer disease?. Nat. Rev. Neurol..

[62] (2015). Alzheimer’s role of breast-cancer gene. Nature.

[63] Beal E. (2010). Cancer link to Alzheimer disease, but not vascular dementia. Nat. Rev. Neurol..

[64] Majd S., Power J., Majd Z. (2019). Alzheimer’s Disease and Cancer: When Two Monsters Cannot Be Together. Front. Neurosci..

[65] Koppenol W.H., Bounds P.L., Dang C.V. (2011). Otto Warburg’s contributions to current concepts of cancer metabolism. Nat. Rev. Cancer.

[66] Demetrius L.A., Magistretti P.J., Pellerin L. (2014). Alzheimer’s disease: The amyloid hypothesis and the Inverse Warburg effect. Front. Physiol..

[67] Demetrius L.A., Simon D.K. (2012). An inverse-Warburg effect and the origin of Alzheimer’s disease. Biogerontology.

[68] Chen Z., Liu M., Li L., Chen L. (2018). Involvement of the Warburg effect in non-tumor diseases processes. J. Cell Physiol..

[69] Warburg O. (1956). On the origin of cancer cells. Science.

[70] Atlante A., de Bari L., Bobba A., Amadoro G. (2017). A disease with a sweet tooth: Exploring the Warburg effect in Alzheimer’s disease. Biogerontology.

[71] Zebhauser P.T., Berthele A., Goldhardt O., Diehl-Schmid J., Priller J., Ortner M., Grimmer T. (2022). Cerebrospinal fluid lactate levels along the Alzheimer’s disease continuum and associations with blood-brain barrier integrity, age, cognition, and biomarkers. Alzheimers Res. Ther..

[72] Palmer A.M. (1999). The activity of the pentose phosphate pathway is increased in response to oxidative stress in Alzheimer’s disease. J. Neural. Transm..

[73] Bermejo P., Martín-Aragón S., Benedí J., Susín C., Felici E., Gil P., Ribera J.M., Villar A.M. (2008). Peripheral levels of glutathione and protein oxidation as markers in the development of Alzheimer’s disease from Mild Cognitive Impairment. Free Radic. Res..

[74] Ivanov A.I., Malkov A.E., Waseem T., Mukhtarov M., Buldakova S., Gubkina O., Zilberter M., Zilberter Y. (2014). Glycolysis and oxidative phosphorylation in neurons and astrocytes during network activity in hippocampal slices. J. Cereb. Blood Flow. Metab..

[75] Liu F., Shi J., Tanimukai H., Gu J., Gu J., Grundke-Iqbal I., Iqbal K., Gong C.X. (2009). Reduced O-GlcNAcylation links lower brain glucose metabolism and tau pathology in Alzheimer’s disease. Brain.

[76] Rawal P., Zhao L. (2021). Sialometabolism in Brain Health and Alzheimer’s Disease. Front. Neurosci..

[77] Moon H.J., Haroutunian V., Zhao L. (2022). Human apolipoprotein E isoforms are differentially sialylated and the sialic acid moiety in ApoE2 attenuates ApoE2-Aβ interaction and Aβ fibrillation. Neurobiol. Dis..

[78] Robey R.B., Hay N. (2006). Mitochondrial hexokinases, novel mediators of the antiapoptotic effects of growth factors and Akt. Oncogene.

[79] Pastorino J.G., Shulga N., Hoek J.B. (2002). Mitochondrial binding of hexokinase II inhibits Bax-induced cytochrome c release and apoptosis. J. Biol. Chem..

[80] Chiara F., Castellaro D., Marin O., Petronilli V., Brusilow W.S., Juhaszova M., Sollott S.J., Forte M., Bernardi P., Rasola A. (2008). Hexokinase II detachment from mitochondria triggers apoptosis through the permeability transition pore independent of voltage-dependent anion channels. PLoS ONE.

[81] Abu-Hamad S., Zaid H., Israelson A., Nahon E., Shoshan-Barmatz V. (2008). Hexokinase-I protection against apoptotic cell death is mediated via interaction with the voltage-dependent anion channel-1: Mapping the site of binding. J. Biol. Chem..

[82] Miura T., Tanno M. (2012). The mPTP and its regulatory proteins: Final common targets of signalling pathways for protection against necrosis. Cardiovasc. Res..

[83] da-Silva W.S., Gómez-Puyou A., de Gómez-Puyou M.T., Moreno-Sanchez R., De Felice F.G., de Meis L., Oliveira M.F., Galina A. (2004). Mitochondrial bound hexokinase activity as a preventive antioxidant defense: Steady-state ADP formation as a regulatory mechanism of membrane potential and reactive oxygen species generation in mitochondria. J. Biol. Chem..

[84] Chen W.Y., Chen Y.F., Chan H.C., Chung C.H., Peng H.Y., Ho Y.C., Chen C.H., Chang K.C., Tang C.H., Lee A.S. (2020). Role of apolipoprotein E in electronegative low-density lipoprotein-induced mitochondrial dysfunction in cardiomyocytes. Metabolism.

[85] Zhao L., Gottesdiener A.J., Parmar M., Li M., Kaminsky S.M., Chiuchiolo M.J., Sondhi D., Sullivan P.M., Holtzman D.M., Crystal R.G. (2016). Intracerebral adeno-associated virus gene delivery of apolipoprotein E2 markedly reduces brain amyloid pathology in Alzheimer’s disease mouse models. Neurobiol. Aging.

[86] Hudry E., Dashkoff J., Roe A.D., Takeda S., Koffie R.M., Hashimoto T., Scheel M., Spires-Jones T., Arbel-Ornath M., Betensky R. (2013). Gene transfer of human Apoe isoforms results in differential modulation of amyloid deposition and neurotoxicity in mouse brain. Sci. Transl. Med..

[87] Theendakara V., Peters-Libeu C.A., Bredesen D.E., Rao R.V. (2018). Transcriptional Effects of ApoE4: Relevance to Alzheimer’s Disease. Mol. Neurobiol..

[88] Parcon P.A., Balasubramaniam M., Ayyadevara S., Jones R.A., Liu L., Shmookler Reis R.J., Barger S.W., Mrak R.E., Griffin W.S.T. (2018). Apolipoprotein E4 inhibits autophagy gene products through direct, specific binding to CLEAR motifs. Alzheimers Dement. J. Alzheimers Assoc..

[89] Zhu J., Li P., Zhou Y.G., Ye J. (2020). Altered Energy Metabolism During Early Optic Nerve Crush Injury: Implications of Warburg-Like Aerobic Glycolysis in Facilitating Retinal Ganglion Cell Survival. Neurosci. Bull..

